# Selling health and happiness how influencers communicate on Instagram about dieting and exercise: mixed methods research

**DOI:** 10.1186/s12889-019-7387-8

**Published:** 2019-08-06

**Authors:** Katharina Pilgrim, Sabine Bohnet-Joschko

**Affiliations:** Chair of Management and Innovation in Health Care, Witten/Herdecke University, Witten, Germany

**Keywords:** Instagram, Public health, Social media, Health communication, Influencer, Eating disorder, Prevention, Fitspiration

## Abstract

**Background:**

Eating disorders among adolescents are an ongoing public health concern. Sustainable health promotion programmes require a thorough understanding of the social context in which minors engage. Initial studies show that young people make extensive use of social networks in order to exchange experiences and gather information. During this process their (buying) behaviour is significantly affected by so-called influencers.

**Methods:**

The exploratory research studies non-campaign driven health communication on dieting and exercise by influencers in social networks with a focus on content, techniques and visible impact. In a mixed methods approach, we initially analysed 1000 posts from influencers on Instagram quantitatively. Subsequently we conducted an in-depth content analysis of 9 extreme and 27 typical communication threads.

**Results:**

Influencers gain the trust and friendship of their followers by designing body-shape focused visual content and targeted communication techniques. They identify and define diet and exercise as factors to be controlled for body perfection. By consuming dietary supplements and wearing tight-fitting branded sportswear, influencers promise a simplified way of optimizing one’s appearance as the key to happiness. Direct and surreptitious advertising of industry-specific products constitutes the communicative focus. At the same time, minors identify with the roles and ideals demonstrated by influencers and their needs are satisfied on several different levels. This creates a relationship of dependency between influencers and their followers.

**Conclusions:**

The dynamics in the field of health communication by influencers on social networks will become increasingly important in the coming years. This is largely due to the targeted demand on the part of (mainly) underage users and the high attractiveness of influencer marketing on the part of companies. Influencers suggest a dependence on happiness, well-being, health and beauty. Only those who create a body shaped through control and discipline are healthy and beautiful - and can be happy. The indirectly communicated conclusions, which can be considered as extremely critical, illustrate the need for action in order to protect and positively accompany young people in their psychological and physical development. The shift of authority figures within Generation Z, as well as identified communication techniques, can be considered and may be harnessed by targeted, group-oriented campaign designs.

**Electronic supplementary material:**

The online version of this article (10.1186/s12889-019-7387-8) contains supplementary material, which is available to authorized users.

## Background

Numerous studies have confirmed unhealthy individual dietary habits and lack of exercise as risk factors for the development of certain chronic diseases [1–3]. Since habitual unhealthy behaviours during childhood and adolescence are often carried into adulthood, the high rate of morbid over- or underweight minors continues to be a public health concern [4]. The number of overweight or obese young children increased from 32 million globally in 1990 to 41 million in 2016 [5]. If current trends continue that number will reach 70 million by 2025 [5]. The consequences of morbid obesity during childhood and adolescence include an increased risk of high blood pressure as well as glucose metabolism disorders [2]. In Germany, 15.4% of all girls and boys aged between 3 and 17 are overweight [6]. At the same time, 20% of 11- to 17-year-olds have an eating disorder with boys and girls affected comparably [6]. Being underweight as a result of unhealthy eating habits in adolescence, can lead to multiple, sometimes irreversible, physical conditions such as osteoporosis, atherosclerosis or even death [1].

An understanding of children’s behavioural and communication patterns is essential for the efficient prevention of obese and morbidly underweight children and adolescents, as well as the emergence of related diseases [7]. Identifying existing channels for collecting, sharing and exchanging information is the key to success in implementing sustainable health promotion measures [8]. Campaigns are more likely to change attitudes if they understand the target audience and tailor messages to specific target audience characteristics [9]. In order to reach young adults, online interventions play an increasingly important role in the design of health campaigns [10]. Online relationships developed through social networks can strengthen behavioural norms and establish common interests and trust [4]. International research indicates that children and adolescents are most likely to access and engage with health information online through popular social networking websites [11]. A national panel (Germany) indicated that the vast majority (85%) of 12- to 17-year-olds spend almost 3 hrs (166 min) per day on social networks [12]. In this context, so-called influencers, individuals who influence an exceptionally large number of their peers, form standards of orientation [13, 14]. Currently more than one-third of 14- to 17-year-olds are deliberately looking for products and services on influencer’s accounts [14]. Instagram has established itself as the most important social network for influencers as it enables the most effective interaction with their own network and is the best channel for paid collaboration [15]. The network has been growing disproportionately fast since its launch in 2010, especially in Germany [16]. In October 2016, Instagram had approximately 6.7 million registered users but by August 2017 their user numbers had jumped to 15 million. This corresponds to a growth rate of 124% over a 9 month period [17]. Referring to demographic user data in Germany, 8.5 million Instagram users are between the ages of 13 and 24 [18]. Looking at the 2015 population of 10 million 13- to 25-year-olds in Germany, more than 85% of German youths use Instagram, with the trend rising [19].

The study aims to contribute to a better understanding of the health-related communication characteristics of influencers in social networks. Our goal is to provide access to a form of health communication at the interface between health promotion and brand communication that has hardly been considered by researchers so far. Content communicated by influencers and communication techniques used, as well as the propagated body image and the role of health were analysed. In addition, we gathered, evaluated and classified digital responses to content communicated by influencers in order to better understand the changing communication behaviour of young people regarding health-related issues.

## Methods

This study used a non-experimental cross-sectional research design. We applied a mixed methods approach. The sequential two-phase design begins with a quantitative study, followed by a qualitative one [20, 21]. The qualitative sample depends on the findings of the quantitative analysis [22]. The integration of both studies takes place at several stages (data collection, analysis and interpretation) [22]. The entire design is determined by an explicit theoretical perspective of relevant communication models [23–25], theories of behavioural change [26–28] and theories of influence [29–32]. For this research study we used quantitative content analysis as well as summarising qualitative content analysis.

### Materials

The research area covers contents of the computer-aided social network Instagram. The starting point of the analysed material, which is available in digital form as a character string (letters, numbers, special characters, emoticons, etc.), is information encoded by binary characters (bits) [33]. Due to the increasing use by and special importance of Instagram to young people, we chose to use this social network as the subject of this study. The audio-visual microblog is an Internet-based application that supports networking, communication between users and the creation and publication of user-generated content [34]. The research area comprises only secondary data (publicly accessible communication threads with the starting point in a picture with caption), which is why an ethics proposal can be waived. We omitted automated computer-aided analysis for both quantitative and qualitative analysis. In the context of a non-automated analysis, we were able to access the online digital research database InfluencerDB. InfluencerDB uses the official Instagram Application Programming Interface (API) and includes every Instagram account with at least 15.000 followers worldwide [35]. Via a premium account we were able to access this dynamic database for research. This included an individually defined search as well as a measurement of the search results on an ordinal scale, for example according to the total number of followers.

### Sample

In May 2018, we ranked the accounts that publish posts in German according to the total number of followers, excluding brand and company profiles.

For the sample definition, relevant accounts communicating on nutrition and exercise needed to be identified. In order to generate a sense of the wording used on Instagram in this context, the research team examined the 100 most frequently used hashtags worldwide [36].

We identified the following hashtags as being relevant and employed them as keywords for searching in account names and profile descriptions:

- gym, fit, fitness, sport, nutrition, train, food.
- Accounts were screened manually.

The sample for the qualitative content analysis was comprised of the top 50 accounts of individuals (by total number of followers) who publish information in German and whose account name or description contains one of the defined keywords. The 50th account selected had a count of 100.000 followers. In context of influencer-marketing, accounts with less than 100.000 followers are categorized as micro-influencers. Since our research aims to derive results for the most influential accounts (regarding follower counts) we chose all relevant accounts with 100.000 followers and more. The sample consisted of 8 male and 42 female influencers regarded by the defined screening criteria.

**Fig. 1:**
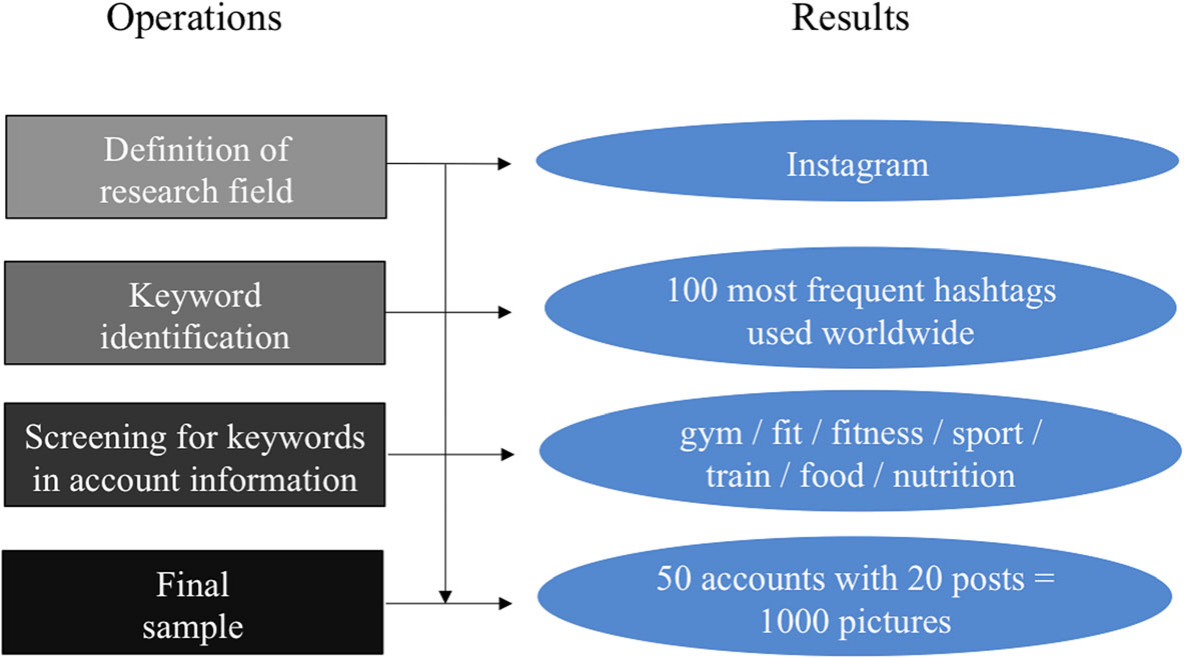
Flow chart of quantitative sample selection

The last 20 posts within each account, showing either food or sportswear, made up the final sample.

(*n* = 1000). In order to assure the up-to-dateness of the data as quality criteria, the selection of newest posts is suitable for our research. The included postings, with the defined characteristics, were posted over different total periods from 5 to 40 weeks, eliminating the selection of newest posts as a source of bias. Figure 1 shows a flow chart of the quantitative sample selection.

We coded 38 items for content analysis. An additional pdf file shows this in more detail (see Additional file 1).

In a next step, the codebook was subdivided into three analysis levels: content, techniques and effects. Using Microsoft Excel and the Statistical Package for the Social Sciences (SPSS) software, quantitative results in the form of frequencies, partial frequencies and correlations were generated.

Based on the frequencies, we selected cases for qualitative content analysis. A case was defined as a caption being the starting point of the digital interaction between influencer and followers, as well as any comments made by followers on the caption or content of the picture shared. The sample size was not determined by a quota but rather it was based on theoretical saturation. The final 27 cases split up in to 18 extreme and 9 typical cases based on the results of the qualitative analysis [37]. For example, one of the items in the quantitative codebook counted the types of food displayed in one picture. If you rank all types of food identified from being displayed least often to most often, processed food was displayed most and was therefore defined a case (maximum) in the qualitative sampling plan. Tables 1 and 2 summarise the selected features and their attributes.

**Table 1:** Quantitative content analysis data on a cardinal scale, creating cases 1 to 15 for qualitative content analysis

	Cases for qualitative content analysis		
	Typical	Extreme	
Feature of the quantitative content analysis	Average	Minimum	Maximum
Like-rate	(1) 4.1%	(2) 0.24%	(3) 23%
Comment-rate	(4) 0.1%	(5) 0.0016%	(6) 2.5%
Number of hashtags	(7) 6	(8) 0	(9) 30
Number of displayed naked body parts	(10) 2	(11) 0	(12) 5
Number of integrated brands	(13) 1	(14) 0	(15) 6

**Table 2:** Quantitative content analysis data on an ordinal scale, creating cases 16 to 27 for qualitative content analysis

Feature	Cases		
	Typical	Minimum	Maximum
Types of displayed food by frequency	(16) Food supplements	(17) Unprocessed	(18) Processed
Types of displayed naked body parts by frequency	(19) Chest	(20) Bottom	(21) Arm
Segments of integrated brands on the subject of diet by frequency	(22) Spices	(23) Chocolate	(24) Food supplements
Segments of integrated brands on the subject of exercise by frequency	(25) Gym	(26) Personal Training	(27) Sportswear

We interpreted the research material obtained from scientific document analysis qualitatively using the MAXQDA software to gain a deeper understanding of the communicated contents, the propagated body image and the communication techniques used. Digital responses by third parties (their followers) were examined and classified in detail. In addition, we have analysed the role of health in the context of communicated content concerning diet and exercise. We abstained from illustrative quotes as the analysed content is in German, mostly “textspeak” and with liberal use of emoticons.

User-generated content on Instagram is classified as a narrative, visual and non-research-generated document and can therefore be used as a source of information in the context of archive research.

Reflecting analysis techniques in the field of qualitative social research, the qualitative content analysis according to Mayring was chosen. The process model according to Mayring is designed to simplify the starting material [38] and is therefore suitable for the large amount (8.089 comments) of material available within the scope of this research study. The aim is to work out the meanings of the selected communication strands in nine stages by means of a data-controlled, step-by-step coding. The individual case turns into a collection of characteristic features. The aim of the summary as an analytical technique is the systematic reduction of qualitative data sets to main contents, with abstraction creating a manageable corpus of data that still maps the basic material [39].

## Results

Based on theoretical models and the current state of research, the analyses carried out show the special importance of influencers in Germany when it comes to health-related topics such as nutrition and exercise. From a public health perspective, influencers pursue non-campaign-driven health communication. At a rate of 84%, it is predominantly female influencers who communicate about diet, nutrition and physical activity. Regimented food intake and strict exercise routines are understood and used as means to achieve and/or maintain a clearly defined body image.

### Communicated content

The focus on fitness, bodybuilding and strength training (or ‘fitspiration’) is clearly illustrated by the staging of the pictures and by large parts of the captions. Influencers present themselves in a gym or provide insight into their past and/or future workout plans. This usually includes training as an essential part of their routine. Fitness and strength training goals are strongly linked to the desire for muscle building and fat reduction, which contribute to a visual and actively controllable shaping of the perceived body image. The presentation of achieved physical goals on the basis of outlined ideals is also strikingly staged. The influencers themselves, dressed in advertised close-fitting sportswear, are the focus of the shared images. Moreover, at this point we cannot trace whether subsequent digital image editing artificially altered the body image. Both pictures and captions suggest a fiction of external perfectionism. Regardless of the season and the topic communicated, influencers propagate a standardized body image: almost 90% of the analysed contributions show influencers with at least one exposed body part. The exposure of arms, legs, back, chest, abdomen or buttocks serves the depiction of the ideal body shape. Active body shaping is focused on targeted muscle building and is also seen as a status symbol. Nearly 90% of the posts with exposed skin show pronounced musculature in the arm area and two-thirds show body core musculature.

At the same time, muscle growth is only visible with a low percentage of body fat thanks to a controlled and optimized diet and daily strength training. This creates a visible, three-dimensional surface relief that supports a measurable shaping of the body. Fitness success is not measured by physical performance, but exclusively by visual appearance.

In this context, the business models of influencers play a special role: 71% of the data examined show at least one brand. 88% of the brands illustrated relate to diet and exercise. In addition, 90% are mentioned by name and directly linked. Brands assigned to the nutrition division can be divided into 15 segments, with food supplements being the most advertised segment (75%). At 80%, the sportswear segment has the largest share in connection with exercise. Concurrently, only half of all contributions with integrated trademarks are labelled as advertising.

### Communication techniques

We were able to identify three categories of communication goals that had to be clearly distinguished from each other. On the one hand, influencers try to position themselves as experts by rhetorical means and specifically selected content. The goal is to increase the total engagement on the account. On the other hand, the majority of observed communication was intended to increase the personal appeal (of the influencer) in the eyes of the followers. The influencer consciously intended to create or increase perceived similarity between her/him and her/his followers, as well as perceived familiarity and sympathy. Table 3 gives an overview of the objectives and techniques used.

**Table 3:** Communication goals and techniques

Generating revenue				
Positioning of advertising		Increasing attractiveness & trustworthiness		
Positioning as expert	Generating commitment	Similarity	Familiarity	Sympathy
Sharing expert information	Activation by an asking a question	Representing socially accepted attitude	Creating intimacy by retelling experiences in the form of diary entries	Presenting positive support through motivating statements
Showcasing personal experience	Activation through self-promotion	Answering a self-asked question	Conveying closeness through personal address	Thanks and approval by direct response to comments

Embedded trademarks, both in images and in image captions, are declared to be a personal preference irrespective of any labelling.

Influencers present brands or products and share their advantages and/or their personal affinity for them. At no time, however, they address the underlying business model, which is therefore not discernible to followers. Personal goals pursued by influencers in the context of communication on diet and exercise should be validated or verified by subsequent research.

### Communicative effects

In order to illustrate the effects of communicated content and identified communication techniques, we have analysed the data used so far, as well as the entire communication threads of the selected 27 cases (*n* < 1000).

We divided the comments into nine categories and assigned them to three different comment types. Both categories and types can be differentiated according to intensity (frequency) and degree of personal reference (proximity between influencer and follower).

**Fig. 2:**
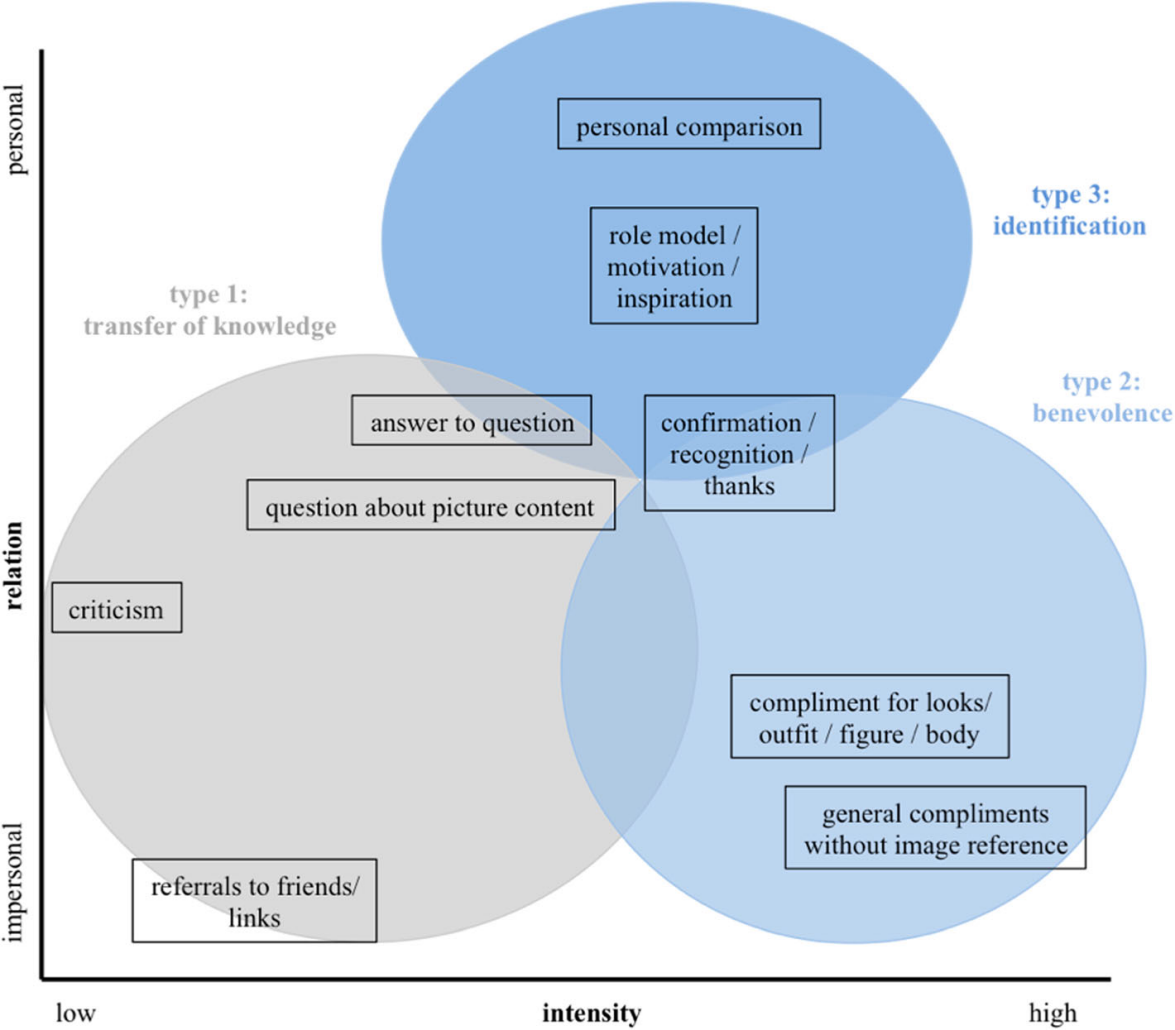
Communicative effects

Type 1 (*knowledge transfer*) includes the four categories *criticism, recommendation to friends, answers to questions* and *questions about image content*. Type 2 (*benevolence)* covers the categories *respect/praise, compliment for appearance/outfit/figure/body, general compliments without image reference* as well as the category *approval/recognition/thanks* (Fig. 2). Comments with the most characters and strongest references are aimed at the *personal identification of followers* (type 3) with the influencer and include the categories *personal comparison* and *motivation/inspiration*. Followers reveal very intimate details about their personal circumstances and express trust and perceived familiarity. They deliberately ask influencers who are perceived as friends for advice, react promptly to the incentives used by influencers and take their recommendations seriously. In this context, a possible upward comparison with benchmarks, perceived as unattainable, can lead to dissatisfaction. On the other hand, social comparison may increase the intrinsic motivation of followers, thus having a positive effect on the process of self-improvement in terms of physical activity and eating habits.

### Communicative process

By using carefully designed images and targeted communication techniques, influencers gain the trust and friendship of their followers in order to strengthen their own brand identity through maximum credibility. At the same time, influencers equate cognitive health with the achievement of a beauty ideal within Generation Z, that includes visible muscles/curves and a low percentage of body fat. Daily physical stimuli and a targeted and planned diet lead to a visible shaping of one’s own body.

Achieving the propagated body ideal through external optimization shall lead to perceived beauty. Bodies created and shaped by control imply self-determination and a fiction of mental health. This cognitive situation is represented by several codes as self-realization through the experience of happiness.

**Fig. 3:**
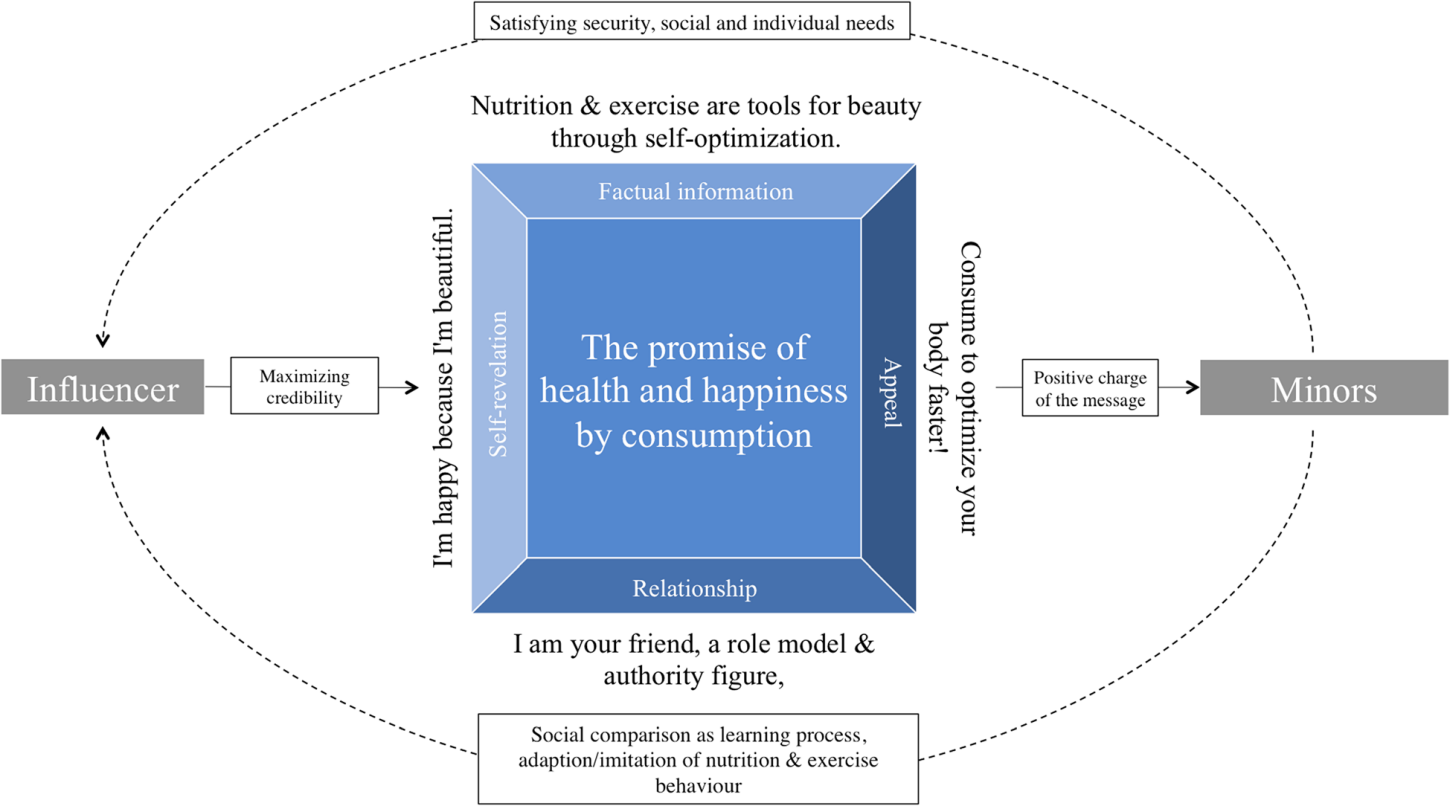
Communicative process

Influencers in their role as brand ambassadors, advertisers and cooperation partners, promote the chance to achieve the ideal body goal described, along with mental health and happiness, through the targeted consumption of dietary supplements and sportswear. At the same time, by pursuing and imitating the strategy to achieve physical, social and cognitive goals, various human needs are met (Fig. 3).

## Discussion

Building a connection between external beauty and perceived well-being, and thus mental health, is a direct effect of influencers’ health communication. Messages imply that an ideal body image cannot be achieved without following the given advice, which predominantly pushes the consumption of presented goods. An alleged dependence on happiness, contentment and beauty is suggested: Only those who are beautiful can achieve happiness. These implicit conclusions illustrate the need for action to protect and positively accompany young people in their psychological and physical development. Understanding the extent to which communicated contents contribute to the promotion or minimisation of eating disorders, obesity or the development of new mental diseases (e.g. orthorexia nervosa), will need further quantitative and qualitative research.

Recent research shows that young women with low self-esteem, depression, the urge for perfectionism and being thin as an ideal of beauty are exactly those user groups who feel more attracted to social networks in order to experience confirmation and satisfy their personal need for security [40, 41].

Previous studies also provide helpful insight into the use of social networks as a tool for positive change in personal eating habits or body image. Social media channels are well suited as nudging tools with the aim of changing behaviour. The more social media content promotes identification, the more it is perceived as realistic. By containing meaningful images and highlighting the perceived parallels between sender and recipient, it has the power to influence users and shift perception [9, 42]. Our results suggest that nutrition and movement content is received at a higher rate than the integrated advertising message. Products presented are commented on or scrutinised to a relatively negligible extent. The focus of observable reactions is on the person and personality of the influencer. Her (or his) figure and clothing, physical assets, and life story/history, are of special interest to followers. Our findings support results of previous research and call attention to Instagram’s effects on health behaviour [43, 44]. Propagating esteem for one’s own person, especially through influencers, may offer an innovative way of counteracting the negative effects that social media has on the contentedness of young women.

### Limitations

There are some limitations to content analysis of Instagram-based data. Abbreviations, neologisms, mixed languages and an incomplete syntax characterize online conversations [45]. This prevents automatic content analysis by programs or may lead to misinterpretations. Furthermore, there is no way to verify the accuracy of the user information. Instagram users may change their location, choose a different language, or provide incorrect information (e.g. their age) [46]. The absolute numbers of followers, likes and comments must also be assessed as critical; a high interaction rate and large coverage increase the market value of an influencer [34]. For this reason, different strategies can be used to artificially increase key figures and supposed popularity (ibid.). So-called fake followers (also known as ghost followers) can be purchased from third-party providers (ibid.). In addition, it is possible to manipulate the number of likes and comments with the help of automated chat bots (ibid.).

### Chances and risks

Social media and particularly the accounts of influencers create a relevant environment for young people. They satisfy needs by establishing intimate relationships and do not only influence buying behaviour, but also social behaviour. By minimizing divergence losses and maximizing viral distribution of content, influencers create the ability to implement and deliver health-promoting programmes directly through their social media channels. Social media campaigns that leverage the interactive strengths of social networking sites have the potential to affect the beliefs and attitudes of the target audience. Regulatory issues such as the lack of information transparency and accuracy, illustrate the risks and challenges of non-campaign-driven health communication by an influencer on diet and exercise.

By using trusted communication techniques and a presentation that is non-transparent (due to the omission of markings), it is a challenge for adolescents and young adults to distinguish between commercial and personal statements. The influencer constitutes surreptitious advertising by omitting advertising markings. From a legal standpoint, there is widespread uncertainty about the labelling requirements for commercial or editorial contributions in social networks, in particular Instagram. Current national laws and regulations have not been adapted to fit the needs of digital communication and still concentrate primarily on mass media such as television, radio and simple websites. It is therefore necessary to establish guidelines from a political point of view or to harmonise existing laws in order to increase the transparency of information for both influencers and followers.

## Conclusion

Specific ideals of body shape and the behaviour propagated in order to achieve these ideals, as well as the shift in authority and attachment figures of minors must be taken into account when developing future strategies of prevention and health promotion. This strategy will make a more positive contribution to the improvement of public health. The dynamics in the field of health communication by influencers in social networks will continue to gain in importance over the next few years. This is mainly due to the targeted demand of predominantly underage users and the high attractiveness of influencer marketing from a commercial point of view.

In addition to regulatory issues, public health professionals, teachers, guardians and decision-makers need to enhance their digital skills to provide minors with appropriate information. In the future, it will be important to understand social media as a setting in which children and young people learn, play, love, create and live according to their own health logic. This platform should be utilised in order to develop effective and sustainable health promotion measures and prevention campaigns. The shift in authority and attachment figures within Generation Z outlined in this paper, as well as identified communication techniques, can be used as a basis for targeted, group-oriented campaign design in the context of future strategies. In order to make a more positive contribution to public health, it is important to protect minors from the dangers of possible disorders while they are developing life-long healthy eating and exercise behaviours.

## Additional file

Additional file 1: Qualitative items for coding. Thirty eight coded items for content analysis. (DOCX 70 kb)

## Data Availability

The datasets used and/or analysed in the context of this study are available from the corresponding author on reasonable request.

## Abbreviations

API: Application Programming Interface
DB: Data base
e.g.: Exempli gratia, for example
et al.: Et alii, and others
n: Sample size
SPSS: Statistical Package for the Social Sciences
WHO: World Health Organization
