# Supplementary material for: Selling health and happiness how influencers communicate on Instagram about dieting and exercise: mixed methods research
Source: BMC Public Health. 2019 Aug 6;19:1054. doi: 10.1186/s12889-019-7387-8 (PMC6683418; doi:10.1186/s12889-019-7387-8)
Supplement: Supplementary file 1 — Qualitative items for coding. Thirty eight coded items for content analysis. (DOCX 70 kb) [file 12889_2019_7387_MOESM1_ESM.docx]

**Additional file 1:**

Qualitative items for coding.

38 coded items for content analysis

1. *Total number of followers*
2. *Total number of likes*
3. *Like-rate*
4. *Total number of comments*
5. *Comment-rate*
6. *Number of hashtags*
7. *Display of food (yes/no)*
8. *Specification of displayed food (free text)*
9. *Unprocessed (yes/no)*
10. *Processed (yes/no)*
11. *Supplement (yes/no)*
12. *Display of sports related item (yes/no)*
13. *Person in sportswear (yes/no)*
14. *Display of naked body part (yes/no)*
15. *Display of naked arm (yes/no)*
16. *Display of naked chest (yes/no)*
17. *Display of naked back (yes/no)*
18. *Display of naked bottom (yes/no)*
19. *Display of naked abdomen (yes/no)*
20. *Display of naked leg (yes/no)*
21. *Number of displayed naked body parts*
22. *Display of visible muscles (yes/no)*
23. *Display of person during a sport-related activity (yes/no)*
24. *Display of brands (yes/no)*
25. *Specification of displayed brands (free text)*
26. *Segmentation of displayed brands (exercise/nutrition/other)*
27. *Food supplement (yes/no)*
28. *Healthy food (yes/no)*
29. *Diet programme (yes/no)*
30. *Energy drink (yes/no)*
31. *Sports apparel (yes/no)*
32. *Sporting footwear (yes/no)*
33. *Sports equipment (yes/no)*
34. *Fitness programme (yes/no)*
35. *Advertising labelling (yes/no)*
36. *Tag in picture (yes/no)*
37. *Mention in post (yes/no)*
38. *Self-advertisement (yes/no)*
